# Spent Coffee Grounds Valorization in Biorefinery Context to Obtain Valuable Products Using Different Extraction Approaches and Solvents

**DOI:** 10.3390/plants12010030

**Published:** 2022-12-22

**Authors:** Maris Lauberts, Inese Mierina, Matiss Pals, Mohammed Ammar Abdul Latheef, Andrei Shishkin

**Affiliations:** 1 Latvian State Institute of Wood Chemistry, LV-1006 Riga, Latvia; 2 Institute of Technology of Organic Chemistry, Faculty of Materials Science and Applied Chemistry, Riga Technical University, Paula Valdena Str. 3, LV-1048 Riga, Latvia; 3 Riga Biomaterials Innovations and Development Centre of RTU, Institute of General Chemical Engineering, Faculty of Materials Science and Applied Chemistry, Riga Technical University, Pulka 3, K-3, LV-1007 Riga, Latvia

**Keywords:** extraction, spent coffee grounds, coffee oil, extraction solvents, total phenolic, value added products

## Abstract

The valuable products that can be isolated from spent coffee ground (SCG) biomass consist of a high number of bioactive components, which are suitable for further application as raw materials in various production chains. This paper presents the potential value of the SCG obtained from large and local coffee beverage producers, for the production of valuable, biologically active products. Despite its high potential, SCG has not been utilized to its full potential value, but is instead discarded as waste in landfills. During its decomposition, SCG emits a large amount of CO_2_ and methane each year. The main novelty of our work is the implementation of sequential extraction with solvents of increased polarity that allows for the maximal removal of the available extractives. In addition, we have compared different extraction techniques, such as conventional and Soxhlet extraction, with more effective accelerated solvent extraction (ASE), which has seen relatively little use in terms of SCG extraction. By comparing these extraction methods and highlighting the key differences between them in terms of extraction yield and obtained extract composition, this work offers key insights for further SCG utilization. By using sequential and one-step accelerated solvent extraction, it is possible to obtain a significant number of extractives from SCG, with a yield above 20% of the starting biomass. The highest yield is for coffee oil, which is obtained with n-hexane ranging between 12% and 14% using accelerated solvent extraction (ASE) according to the scheme: n-hexane→ethyl acetate→60% ethanol. Using single-stage extraction, increasing the ethanol concentration also increases the total phenolic content (TPC) and it ranges between 18.7–23.9 Gallic acid equivalent (GAE) mg/g. The iodine values in the range of 164–174 using ASE and Soxhlet extraction shows that the hexane extracts contain a significant amount of unsaturated fatty acids; coffee oils with a low acid number, in the range of 4.74–6.93, contain few free fatty acids. The characterization of separated coffee oil has shown that it mainly consists of linoleic acid, oleic acid, palmitic acid, stearic acid and a small number of phenolic-type compounds.

## 1. Introduction

Coffee is one of the world’s most popular beverages and one of the most important agricultural crops, globally. Instant coffee manufacturing, as well as coffee brewing, generates a large amount of solid residue (spent coffee grounds, or SCG) that is mostly under-utilized. On average, one ton of green coffee generates approximately 650 kg of solid residue (spent coffee grounds), and approximately 2 kg of wet SCG is generated for every single kg of soluble coffee produced. More than 90% of SCG is directly sent to landfills without proper composting or valorization [1,2]. The disposal of spent coffee grounds causes quite serious environmental problems. Based on the data published by the International Coffee Organization, global coffee consumption reached 10 million tons in 2020/21 [3]. SCG ending up in landfills, emits 28.6 million tons of CO_2_ eq annually, which is comparable to 10.6 million litres of burned diesel fuel [4,5]. Decomposing spent coffee grounds releases methane into the atmosphere; methane is the second most abundant greenhouse gas and has a global warming potential up to 43 times greater than CO_2_. Based on the world’s large amount of waste and increasing pollution, plans have been developed all over the world on how to act to reduce the negative effects caused by household waste. Under the Paris agreement in 2015, the EU committed to cut greenhouse gas emissions in the EU by at least 40% below the 1990 levels by 2030. In 2021, the target was changed to at least 55% reduction by 2030, climate neutrality by 2050, 27% renewable energy capacity installed for the entire EU energy supply and a 27% improvement in energy efficiency [6,7,8]. It is clear that terms such as reuse, recycling and environmental sustainability are outlined as priorities of the European Union. Taking into account all the above, it is necessary to find optimal ways to use this bulk waste, to introduce it into the production chains of the circular economy using innovative biorefining approaches.

**Figure 1:**
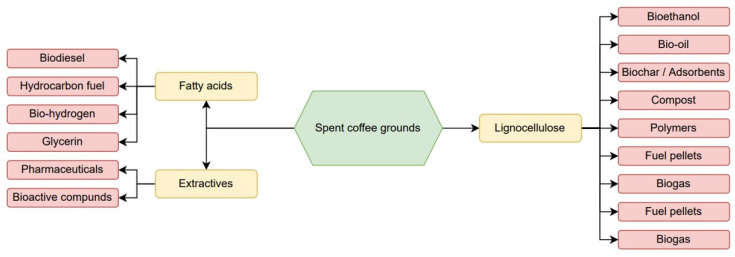
Biorefinery concept of SCG.

Coffee residues have a high potential value to be involved in very wide biorefinery schemes [9,10,11], and the range of products obtained is also very wide, as shown in Figure 1. From a chemical point of view, coffee waste is an inexpensive raw material that contains fatty acids, which could be used as a sustainable carbon source, and it also represents an interesting source of bioactive compounds and fibres. Indeed, coffee residues were proven to be an excellent resource for the production of high-value compounds and energy production [12].

The production of SCG extracts and the purification of individual biomolecules is one of the economically promising stages in the coffee bio-refinery schemes. The multifactorial chemical and biological activities of the extracts obtained from SCG make them prospective raw material for application in the different fields, including: materials production, agriculture, food industry, health care, cosmetic and pharmacology as well. SCGs also contain a significant amount of oil, with over 15% of dry mass, depending on the cultivar of the coffee plant [13,14]. In the literature [15], Yihao Leow et al. demonstrates that using hexane and ethanol as an extractant from spent coffee grounds with reflux extraction for 24 h obtained yields close to 10%, while when using tetrahydrofuran and acetone, the yield is above 10% of the absolutely dry SCG mass [15]. Similarly, using Soxhlet extraction and microwave-assisted extraction, changing different parameters and different degrees of moisture content, the yield of SCG extraction for coffee oil varies between 8.88–11.54% [16]. The composition of SCG depends on feedstock and its treatment during the production of coffee. In general, SCG on dry matter content up to 20% of oil, up to 18% of proteins, >30% of hemicelluloses, from several to 30% of lignin, cellulose, phenolic compounds, including up to 3% of chlorogenic acids, alkaloids including up to 0.4% of caffeine, that predetermines their more beneficial utilization primarily as raw material for the production of high added-value substances [17,18,19]. The polysaccharides not extracted during the water extraction processes when making coffee for daily use will remain in the residual material in the SCG. The application of enzymes involved in the degradation of plant cell walls is a promising way to obtain larger amounts of extracts and more diverse groups of chemical compounds from SCG [20]. The extracts obtained from SCG with different solvents, such as ethanol and its aqueous solutions and methanol and their aqueous solutions, and extraction methods, such as a Soxhlet, hot pressurized liquid extraction, hydrothermal extraction, contain a total phenolic content in the range of 8–93 mg GAE/g and contain biologically active compounds such as 5-caffeoylquinic acid, 3-caffeoylquinic acid, phenolic acids: such as gallic, caffeic, vanillic, syringic, p-coumaric, ferulic, cinnamic acid, flavonoids, i.e., flavonol (quercetin, quercitrin and rutin), flavan-3-ol ((+)-catechin and (-)-epicatechin), anthocyanidins and flavanones (naringin), an alkaloid (quinine), and other bioactive compounds such as resveratrol and shikimic acid [21,22,23].

The extraction is the first step to obtaining valuable products from SCG. Different extraction methods and solvents can be used to extract the SCG oil and other extracts from SCG. The extraction yield and the dominant compounds depend on the extraction method used. If increased pressure is used in the extraction, it helps the solvent molecules to penetrate deeper into the SCG matrix and the extraction is more efficient. Similarly, the solvent used at high pressure and temperature conditions has different physical properties than at atmospheric pressure. By changing the polarity of the used solvent system, extracts with varying chemical compositions can be obtained. The use of non-polar solvents, such as hexane or dichloromethane, yields lipids with extraction yields varying between 20% and 27%. The obtained lipophilic extracts have desirable properties for further integration into various processing pathways. Although these types of solvents are not “green”, similar results can be obtained when using ethanol as an extraction solvent. By changing the ratio of the ethanol/water mixture as the extraction solvent, it is possible to separate a wide range of compounds. When using pure ethanol, it is predominantly lipophilic compounds that are separated, while by increasing the water content in the solvent, more phenolic type compounds can be obtained [18,24].

This extracted oil can be further processed and used in everyday life. For example, as seen in Figure 1, extracted SCG oil can be transesterified and converted to biodiesel through the process of hydrodeoxygenation, and renewable fuels can be manufactured. As visualized in Figure 1, SCG, which is primarily a waste product, can now be transformed and incorporated into various essential products. To gain the maximum possible benefit, the extracted compounds from SCG are being tested to develop better and more cost-effective products to meet the market demand in the near future [25]. The major goal of this study is to extract the natural products (SCG oil) and polar extracts from spent coffee grounds, using different extraction methods and schemes and different solvents, as well as to characterize the obtained extracts with the aim of obtaining valuable products with certain target properties for their potential use.

## 2. Results and Discussion

### 2.1. Sample Characterization

**Figure 2:**
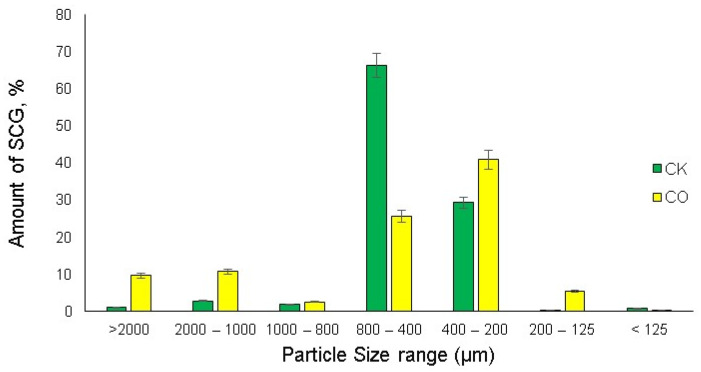
Particle size distribution of CK and CO spent coffee grounds (*n* = 3, error bars indicate SD).

The first step of the sample characterization was to determine the particle size distribution of the spent coffee grounds for the two analyzed samples: CK, from the local large coffee beverage producer, Circle K; CO, from the local large coffee beverage producer, Costa Coffee. The sieving technique was used to find out the particle size distribution of both coffee types, as illustrated in Figure 2.

According to the particle sizes analyzed, most of the CK spent coffee grounds are in the range of 400–800 µm, where around 65% of the particles are settled; on the other hand, most of the particles of CO were settled in the range of 200–400 µm, which is slightly more than 40%. According to the analysis, it is possible to identify that the CO spent coffee ground particles used for the analysis are much finer than the CK SCG particles.

### 2.2. Total Extractive Yields

**Figure 3:**
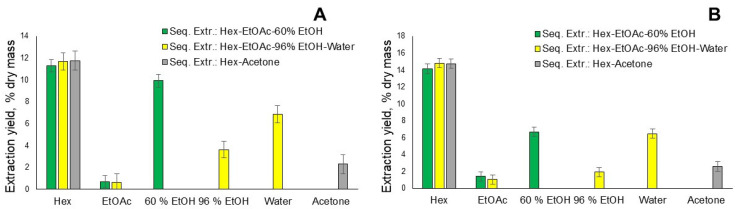
(**A**) Total extraction yield % of CK spent coffee ground sequential extraction with organic solvents, depending on extraction scheme and used solvent. (**B**) Total extraction yield % of CO spent coffee ground sequential extraction with organic solvents, depending on extraction scheme and used solvent (*n* = 3, error bars indicate SD).

The next step was to find out the total extractive yield of the spent coffee grounds using different organic solvents of increasing polarity and water to obtain the different chemical compounds, depending on the polarity of the solvents. When performing the accelerated solvent extraction (ASE) of CK and CO spent coffee grounds, according to the scheme: n-hexane→ethyl acetate→ethanol with accelerated solvent extraction at a temperature of 90 °C degrees for four static cycles, the static time of each cycle was 5 min by changing the ethanol concentration of the last extraction solvent. It can be seen in Figure 3A that the highest total extraction yield, 21.91%, is according to the scheme n-hexane→ethyl acetate→60% ethanol, respectively, 11.28%; 0.7% and 9.93%.

On the other hand, only 15.95% extraction yield is obtained by the scheme n-hexane→ethyl acetate→96% ethanol, respectively, 11.68%, 0.64% and 3.63%. This lowest 96% ethanol extraction yield is explained by the fact that 96% ethanol is less polar than its 60% aqueous solution, and as a result of extraction, more polar groups of SCG do not pass into it than in a 60% ethanol aqueous solution. In order to ascertain the total amount of extractives in the spent coffee grounds, the extraction scheme with 96% ethanol was supplemented as follows: n-hexane→ethyl acetate→96% ethanol→deionised water with another extraction solvent, deionised water, and the obtained results showed that the total extraction yield is 22.81%, as can be seen in Figure 3A. By replacing the extraction solvent ethanol with acetone, it can be seen that after the extraction of n-hexane with acetone, a relatively low yield is obtained, only 2.3% and the total extraction yield is 14.07%. On the other hand, the n-hexane extracts, as a result of sequential extraction, individually show the highest extraction yields, of almost 12% of the starting SCG mass.

The second analyzed SCG sample CO showed slightly different distributions of extraction yields, as can be seen in Figure 3B. The yield of the n-hexane extraction is above 14% of the raw material, but the yield of the 60% ethanol aqueous solution is lower, after the extraction scheme, n-hexane→ethyl acetate→60% ethanol, although the total yield of extractives is 22.22%, which is very close to that of the CK sample after such an extraction scheme. Exactly the same trend, with a reduced yield of 96% ethanol, can be observed for the scheme n-hexane→ethyl acetate→96% ethanol, while according to the scheme where water is taken as the fourth extraction solvent, its yield is 6.46%; this is very close to that in the case of CK, respectively 6.86%, while the total yield of the extractives will be 24.26%.

**Figure 4:**
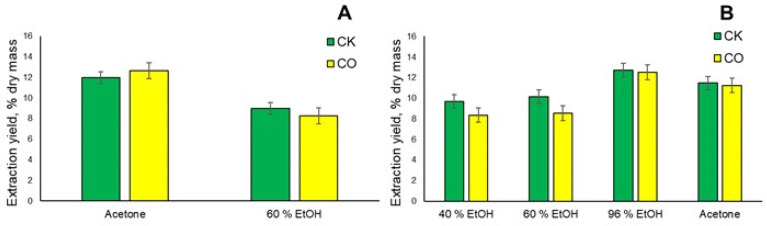
(**A**) Total extraction yield % of spent coffee ground sequential extraction with organic solvents, replacing n-hexane with acetone as the first extraction solvent. (**B**) Total extraction yield % of spent coffee grounds single step extraction with ethanol aqueous solutions in different concentrations and acetone as extraction solvent (*n* = 3, error bars indicate SD).

When performing sequential extraction with ASE according to the scheme acetone→60% ethanol, replacing n-hexane with acetone as the first extraction solvent for the CK sample, it can be seen that the extraction yield is 11.96% and for the CO sample the extraction yield is 12.65% (see Figure 4A); the yield of the 60% ethanol solution is 9.01% and 8.26% respectively, which is very close to the sequential extraction according to the scheme n-hexane→ethyl acetate→60% ethanol and the total extraction yield is 20.97% and 20.91%, respectively. By using acetone, due to its greater polarity compared to n-hexane, in addition to lipophilic compounds, more polar compounds could be extracted.

When performing the single-step extraction of both spent coffee grounds samples, CK and CO, with the accelerated solvent extraction to the same parameters as in the course of sequential extraction, it can be observed that when using 40%, 60% and 96% ethanol solutions in the case of the CO sample, the extraction yields are lower than in the CK sample, as can be seen in Figure 4B.

**Figure 5:**
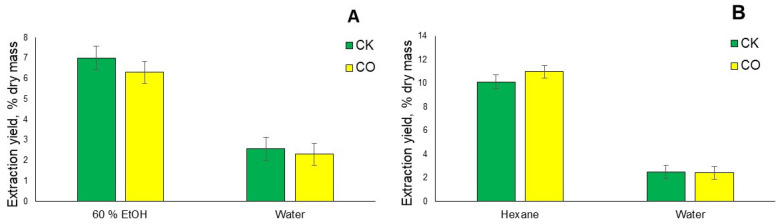
(**A**) Total extraction yield % of spent coffee ground sequential conventional extraction with a reflux condenser. (**B**) Total extraction yield % of spent coffee grounds sequential Soxhlet extraction with n-hexane and water (*n* = 3, error bars indicate SD).

It can be observed that as the ethanol concentration increases, the yield of the extractive substances increases in both of the analyzed SCG samples. When performing extraction with 40% and 60% ethanol solutions, the differences are not drastically large, while increasing the ethanol concentration to a 96% solution increases the yield by 32% in the case of CO extraction. Comparing the results with the works of the other authors, for example, n-hexane and 95% ethanol extracts obtained in a batch process from *Arabica* spent coffee grounds obtained 14.5% and 11.2% yields, respectively, while for the CO sample the n-hexane yield reaches 14.82% (see Figure 3B), which is basically very similar, while the CK sample 96% ethanol extract is obtained with a yield of 12.71% (see Figure 4B), which is slightly more [26]. While performing conventional Soxhlet extraction with n-hexane for 6 h, Chemat et al. showed that spent coffee ground from a cafeteria (CROUS, Agroparc, Avignon) is able to obtain n-hexane extract with a yield of 12.47%, which is comparable to the results obtained in this work [27]. The increased yield of 96% ethanol can be explained by the fact that lipophilic compounds are also extracted from the spent coffee grounds with a solution of this concentration, which is part of coffee oil and is usually extracted with n-hexane, because the obtained extract has an oily consistency compared to the lower concentration of ethanol solutions. By performing a single-step extraction with acetone, it is possible to obtain a result close to the result of the 96% ethanol extract. However, as can be seen in Figure 3A, as a result of successive extraction, its yield was only 2.3%, from which it can be concluded that many more lipophilic compounds are released when n-hexane passes into the acetone extract. In addition, sequential conventional extraction with a reflux condenser was performed to verify the efficiency of the simple extraction and, as can be seen in Figure 5A, the results obtained for the 60% ethanol water extract are lower than in the cases of sequential and single-step extractions with different schemes (Figure 3A,B and Figure 4A,B), and the yield of deionized water is even three times lower. When performing the classical Soxhlet extraction for both analyzed SCG for 6 h at the boiling temperature of the solvents, lower extraction yields were obtained than in the ASE extraction and conventional extraction with a reflux condenser (Figure 5B). The disadvantages of the Soxhlet extraction should also be mentioned, as it is not possible to perform the extraction with the previously used ethanol-water solutions.

It should also be mentioned that when using sequential conventional extraction, one has to face various difficulties, for example, intensive stirring of the extraction cannot be conducted because SCG sediments quickly and local heating occurs.

### 2.3. Total Phenolic Content

**Figure 6:**
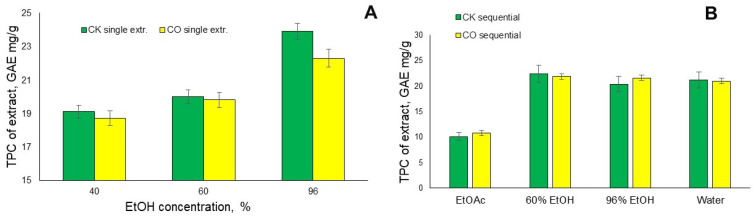
The total phenolic content (TPC) in the extracts isolated from CK and CO spent coffee grounds in the results of it’s (**A**) single step extraction with different ethanol solution concentrations (**B**) sequential extraction with ethyl acetate and 60% and 96% ethanol solution and expressed as GAE mg/g on the isolated extracts (*n* = 3, error bars indicate SD).

As can be seen, the lowest TPC content occurred for both of the analyzed samples CK and CO extracts obtained with ethyl acetate (see Figure 6B), and it is 10.1 and 10.8 mg GAE/g extract, respectively. While in the case of single-step extraction, a trend can be observed, as the ethanol concentration increases from 40% to 96%, the TPC content also increases, as can be seen in Figure 6A.

On the other hand, as a result of sequential extraction, the TPC content of both the CK and CO spent coffee ground extracts is slightly different. The TPC content of the water isolated extracts is practically the same, at 21 mg GAE/g extract, whereas the 60% ethanol extract of CK shows the highest TPC content at 22.4 mg GAE/g, while for the same sample, 96% ethanol extract shows only 20.4 mg GAE/g. Comparing the works of other authors, for example, from SCG collected from bars in the city of Rome with ethanol solutions at different concentrations of extracts, the TPC ranges between 6.33 and 28.26 mg GAE/g extract [28]. In addition, the authors in reference [29] report TPC contents of ethanol extracts from SCG in the range of between 9.23 and 19.49 mg GAE/g extract 2.4. 

The acid values and iodine values of both spent coffee ground hexane extracts obtained with the Soxhlet and ASE methods were determined, as shown in Table 1. The iodine value shows that the hexane extracts contain a significant amount of unsaturated fatty acids.

The hexane extracts separated by the ASE method have a lower content of free fatty acids compared to the extracts obtained during Soxhlet extraction. On the other hand, the iodine numbers of the obtained extracts are quite similar. Comparing the data with the works of other authors, for example, the iodine numbers obtained with supercritical CO_2_ extraction for the SCG extract are half as small, whereas the acid numbers with different solvents, such as pentane, hexane, toluene, and chloroform extracts from SCG, are 7.1; 7.3; 8.3 and 9.1 mg KOH/g, respectively, which are higher than the CK and CO spent coffee ground n-hexane extracts [30,31].

### 2.4. GC-MS Analysis

**Figure 7:**
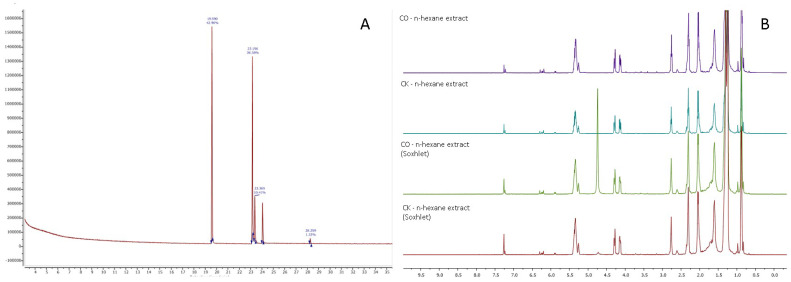
(**A**) CK spent coffee ground n-hexane extract gas chromatography mass spectrometry chromatogram. (**B**) CK and CO spent coffee ground n-hexane extract ^1^H NMR spectra (500 MHz, CDCl_3_).

The obtained n-hexane extracts were analyzed by gas chromatography mass spectrometry, and 4 dominant compounds were identified, as shown in Figure 7A. According to the GC-MS analysis of all the samples, the obtained results are given in Table 2.

### 2.5. NMR Analysis

In order to confirm the results obtained in Table 2, an ^1^HNMR spectra of the obtained hexane extracts were performed (see Figure 7B).

The ^1^H NMR spectra for the hexane extracts is typical for triglycerides: the signals in the range of 0.8–2.8 ppm represent alkyl chains of fatty acids, the doublets at 4.0–4.3 ppm represent the glycerol residue of the triglyceride and the signals at 5.0–5.5 ppm are characteristic of the double bonds of the unsaturated fatty acids and glycerol residue. According to the NMR spectra, the main fatty acid is linoleic acid: the amount varies between 35 and 42%. The amount of oleic acid is 8–12%. Around 30–40% of all fatty acids are saturated. The analysis of the ^1^H and ^13^C NMR spectra shows a significant amount of various other non-triglyceride-type compounds. These minor components might be different phenyl-type compounds, as evidenced by the characteristic signals between 6–7.2 ppm.

**Figure 8:**
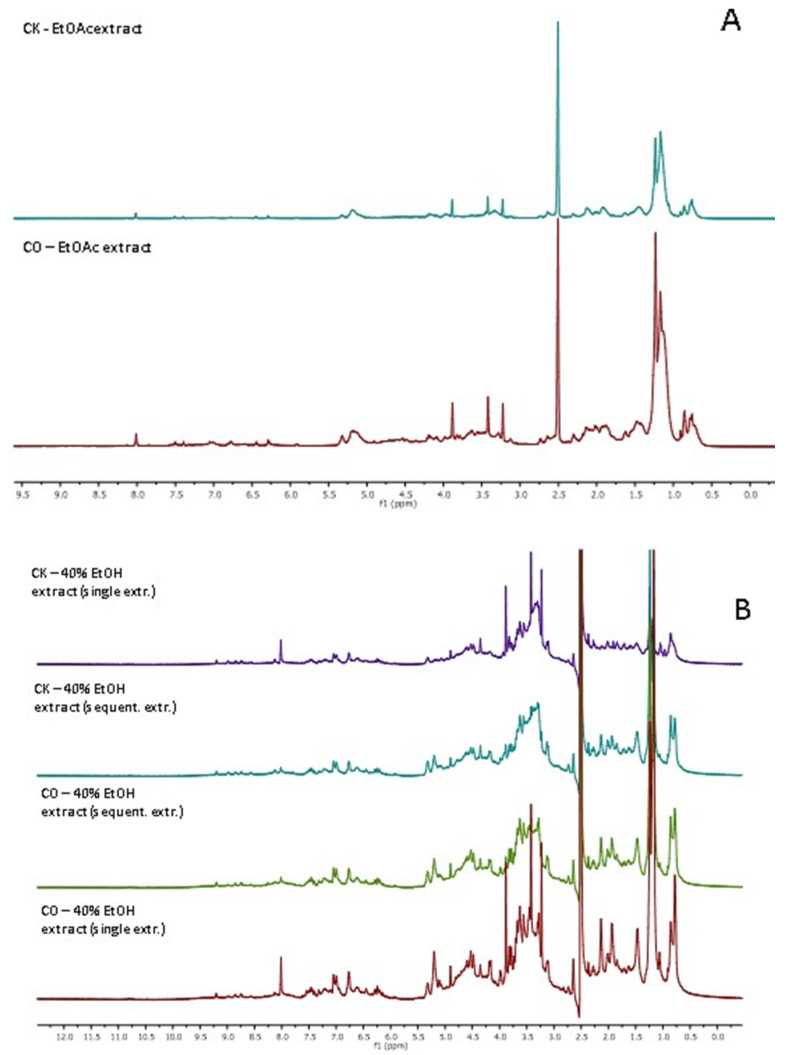
(**A**) CK and CO spent coffee ground ^1^H NMR spectra for ethyl acetate extracts (500 MHz, DMSO-d6). (**B**) CK and CO spent coffee ground ^1^H NMR spectra for 40% ethanol extracts (500 MHz, DMSO-d6).

According to the ^1^H NMR spectra, the ethyl acetate extraction was effective enough for the qualitative removal of triglycerides (signals corresponding to various triglycerides cannot be distinguished). On the other hand, the spectra clearly demonstrate the presence of caffeine in these extracts (characteristic singlets at 8.0, 3.3, 3.5 and 4.0 ppm) (see Figure 8A). 

Regarding the ethanol extracts, the compositions is more complex: instead of various fatty acid derivatives, the signals (0.5–5.5 ppm) characteristic to carbohydrate and peptide residues are present. The signals characteristic to various aromatic compounds are detected at the range of 6–7.5 ppm. However, due to the existence of phenol type compounds in the plants in various forms, the interpretation of the spectra for the crude extracts obtained with polar or medium polar solvents is rather limited, contrary to the spectra of the n-hexane extracts. However, the ^1^H NMR spectra might be an essential tool for the convenient detection of caffeine (see for the characteristic singlets at 8.0, 3.3, 3.5 and 4.0 ppm). The main advantage of the ^1^H NMR over, e.g., classical HPLC analysis, is the simple preparation of the samples: the crude sample is directly dissolved in an appropriate solvent without additional treatment. The ^1^H NMR spectra also clearly indicate that ethyl acetate is an effective solvent for the qualitative extraction of caffeine: those 40% ethanol extracts (see Figure 8B) which were obtained by single-step extraction sequence contain well-assignable signals of caffeine, whilst those extracts which were obtained by the sequential extraction method (after extraction with n-hexane and ethyl acetate) did not contain the corresponding signals (or the signals are so negligible that they are overlapping with other small signals).

## 3. Materials and Methods

### 3.1. Spent Coffee Ground Sample Treatment

The spent coffee grounds (SCG) were provided from local and large coffee beverage producers, Circle K (CK) and Costa Coffee (CO). After collection, the SCGs were dried at room temperature until the moisture content was approximately 10%. The SCG was stored in a freezer at −18 °C until further analysis.

### 3.2. Chemicals and Reagents

The N-hexane, ethyl acetate, methanol, ethanol, Folin–Ciocalteu reagent, gallic acid, Sodium carbonate, potassium hydroxide, diethyl ether, phenolphthalein, starch indicator were purchased from Sigma-Aldrich (Steinheim, Germany). Wijs solution (0.1 mol∙L^−^^1^ iodine monochloride, acetic acid) was purchased from (Fluka (Steinheim, Germany)), chloroform, acetic acid by (lachner), potassium iodide, sodium thiosulfate by (CHEMPUR (Poland)). The methylene chloride, sodium chloride, sodium sulfate were provided by (Acros Organics (Great Britain)). All of the chemicals used for the analyses were of analytical grade, and all of the test solutions were freshly prepared prior to use.

### 3.3. Extraction Procedures

#### 3.3.1. Soxhlet Extraction

Conventional Soxhlet apparatus was used for the Soxhlet extraction technique. The SCG particles were dried in the oven, at 105 °C temperature for 8 h. The cellulose thimbles were also dried in the oven for 8 h before filling them with coffee, about 3/4 of the way, and sealing them with a piece of cotton wool. The n-hexane within 10 g of the dry SCGs were refluxed in a Soxhlet apparatus. The procedure consists of 150 mL of solvent recycling over dried sample, in a Soxhlet apparatus for 6 h extraction at the boiling temperature of the solvent used. After the extraction, the used solvents were evaporated in vacuo (rotary evaporator, Heidolph Instruments (Schwabach, Germany)) and the extracts obtained were stored at −20 °C. All of the results were expressed on a dry weight and ash-free basis.

#### 3.3.2. Accelerated Solvent Extraction (ASE)

Extraction with ASE was accomplished in two ways:

1. Single-step solvent extraction.
2. Sequential extraction with multiple solvents in a sequential order.

#### 3.3.3. Single-Step Solvent Extraction

The extraction cell is filled with dried 36.5 g of SCG and inserted into a Thermo ScientificTM ASE350 extractor and the extraction is carried out with 40%, 60% and 96% EtOH solution as the solvent, at 90 °C for a static time of 5 min and 4 cycles. After the extraction, the used solvents were evaporated in vacuo (rotary evaporator, Heidolph Instruments) and freeze-dried (freeze dryer, Heto PowerDry PL3000 (Allerod, Denmark)), and the extracts obtained were stored at −20 °C. All of the results were expressed on a dry weight and ash-free basis.

#### 3.3.4. Sequential Extraction with Multiple Solvents

The extraction was carried out using the following solvents: n-hexane→ethyl acetate→60% ethanol; n-hexane→ethyl acetate→96% ethanol and n-hexane→ethyl acetate→96% ethanol→deionised water. The extraction was performed at 90 °C for a static time of 5 min and 4 cycles. After the extraction, the used solvents were evaporated in vacuo (rotary evaporator, Heidolph Instruments) and freeze-dried (freeze dryer, Heto PowerDry PL3000), and the extracts obtained were stored at −20 °C. All of the results were expressed on a dry weight and ash-free basis [32,33,34,35].

### 3.4. Analysis of the Extracts

The extracts obtained from the different extraction techniques and using different solvents were used for the analysis, such as total phenolic content (TPC), acid value and iodine value. Furthermore, the extracts were transesterified and GC analysis was conducted to determine the methyl esters which are present in the extracted oils and NMR analysis of the extracts was performed.

#### 3.4.1. Total Phenolic Content

The content of the extractable phenolics (TPC) in the obtained extracts were determined by Folin–Ciocalteu analysis. A volume of 1 mL of hydrophilic extractives solution (in 50% (*v*:*v*) ethanol) was added to 0.5 mL of the Folin–Ciocalteu phenol reagent, followed by gentle shaking. After 5 min, 1 mL of 20% (*w*/*v*) sodium carbonate was added. The solution was immediately diluted up to 5 mL with distilled water and mixed thoroughly. After 10 min, an optical density at 765 nm of the resulting blue complex was measured using a PerkinElmer Lambda 650 UV/VIS spectrophotometer (Llantrisant, GB) against the blank, using gallic acid as the standard. The total phenolic content was expressed as mg of gallic acid equivalents (GAE) per g of dried extract sample [36,37].

#### 3.4.2. Acid Value

0.1 mol·L^−^^1^ KOH in EtOH solution was prepared and this was used as the indicator for the titration. The exact concentration of KOH was determined by titration with 0.1 mol·L^−1^ HCl solution (10 mL) using phenolphthalein as indicator. A solution of EtOH:diethyl ether with a ratio of 1:2 was prepared, and 20 mL were added to the extracted samples and a few drops of phenolphthalein were added to the solution and was titrated against the KOH in EtOH, titrated volume was used to find the acid value of the extracted samples by the following Equation (1):

AV = (V_KOH_ × C_KOH_ × M_KOH_)/m_oil_ (1)

where

AV—acid value, V_KOH_—volume of titration (mL), C_KOH_—concentration of KOH(mol∙L^−^^1^),

M_KOH_—molar mass of KOH (g∙mol^−1^), m_oil_—mass of extracted oil used (g) the unit used for acid value is mg KOH∙g^−1^.

#### 3.4.3. Iodine Value

The extract obtained using hexane as solvent was used to identify the iodine value in the oil samples. Approximately 0.1 g of oil was placed in the flask and 20 mL of chloroform and acetic acid (1:1; *v*:*v*) was added to that, 25 mL of Wijs solution (0.1 mol∙L^−^^1^ iodine monochloride, acetic acid solution) was added, and the mixture was placed in the dark for an hour. Approximately 10 g of KI was dissolved in 100 mL water, and 20 mL of the solution was added to the mixture prepared with it 100 mL water was added. The solution was titrated against 0.1 M Na_2_S_2_O_3,_ and starch was used as indicator. The titration was conducted until the blue solution turned colourless, and the volume used for the titration was used for the calculation of iodine value. A blank solution without the addition of extracted oil were prepared for the calculation of iodine value. The iodine value was calculated using the following Equation (2):

IV = ((V_blank_ − V_sample_)∙C(Na_2_S_2_O_3_)∙M(I_2_))/(m_sample_∙10) (2)

where:

IV—iodine value (mol∙L^−^^1^), V_blank_—titration volume of blank (L), V_sample_—titration volume of sample (L), M(I_2_)—molar mass of iodine (g∙mol^−^^1^), m_sample_—mass of sample used (g) the unit for iodine value is gI_2_∙100 g^−^^1^.

#### 3.4.4. Gas Chromatography Mass Spectrometry (GC-MS) Analysis

The extracts of the oil samples were transesterified (SCG oil is directly esterified with a methanol-2.5% potassium hydroxide (KOH) solution) and then used for the GC analysis. In a 25 mL volumetric flask, 25 mg of transesterified oil was dissolved in hexane. Then, 2–3 µL of the prepared solution was injected into the gas chromatograph mass spectrometer. The chromatogram was run for 30 min, and the peaks obtained were used to determine the methyl esters present in the oil sample.

#### 3.4.5. NMR Analysis

The crude extract (20–50 mg) was dissolved in the appropriate solvent (0.7 mL; CDCl3 for hexane extracts and DMSO-d6 for ethyl acetate and ethanol extracts). Next, ^1^H NMR spectra were recorded on Bruker Avance 500 (Boston, MA, USA)(^1^H: 500 MHz) spectrometer. The spectra were calibrated with respect to the peak of residual solvent (7.26 ppm for chloroform and 2.50 for DMSO).

### 3.5. Statistical Treatment of the Results

The measured values are shown as an average with a confidence interval (at a level of significance α = 0.05). Each measurement was performed at least in triplicate. Statistical calculations were carried out using MS Excel and IBM SPSS Statistics 21.0 (Microsoft Corp, Redmond, WA, USA).

## 4. Conclusions

By sequential extraction with organic solvents, the highest yield of SCG extractives was 24% from dry mass, according to the following scheme: n-hexane→ethyl acetate→96% ethanol→deionised water. By using different polarity solvents, it is possible to divide the extractable compounds from the SCG into groups, to separate the lipophilic compounds (coffee oil) with n-hexane, the semi-polar compounds with ethyl acetate and the polar compounds with ethanol and with water. The extracts obtained with ethyl acetate showed two times lower TPC when compared to the extracts extracted with aqueous ethanol solutions. The highest TPC content of 23.9 mg GAE/g of the extract is obtained by one-step extraction with 96% ethanol solution during ASE extraction. Sequential extraction with acetone does not provide significant benefits, as the first extraction step with the hexane extracts caused most of the acetone-soluble fraction. Conventional extraction under reflux can be used to extract valuable products from SCG; however, with lower extraction yields compared to the ASE method. The coffee oil obtained from SCG with hexane extraction predominantly contained palmitic and linoleic acid, which in total makes up more than 80% of the total extract. Using the determined acid values, it can be concluded that coffee oils contain few free fatty acids. With a high 96% ethanol, an extract is obtained that has a lipophilic part, which could hinder their potential use in hydrophilic substances; on the other hand, it makes it easier to introduce into substances containing oils and fats. In hydrophobic substances, it allows the potential use of these extracts in various fields, that, for example, can be added to cosmetic products as biologically active agents.

## Figures and Tables

**Table 1:** Iodine values and acid value characteristics of n-hexane extracts of CK and CO spent coffee grounds were obtained using ASE and Soxhlet extraction.

Solvent	Iodine Value gI_2_/100 g	Acid Value mgKOH/g
CO—n-hexane extract (ASE)	164.92 ± 4.95	4.74 ± 0.17
CK—n-hexane extract (ASE)	170.28 ± 5.24	5.58 ± 0.19
CO—n-hexane extract (Soxhlet)	170.58 ± 5.53	6.63 ± 0.37
CK—n-hexane extract (Soxhlet)	174.33 ± 4.87	6.93 ± 0.26

**Table 2:** Identified compounds in CK and CO n-hexane extracts from spent coffee grounds used sequential ASE and single-step Soxhlet extraction.

Solvent	Palmitic Acid, %	Stearic Acid, %	Oleic Acid, %	Linoleic Acid, %
CO—n-hexane extract (ASE)	43.1 ± 0.9	8.4 ± 0.2	11.8 ± 0.3	38.1 ± 0.9
CK—n-hexane extract (ASE)	45.1 ± 1.1	9.2 ± 0.2	10.9 ± 0.3	35.0 ± 0.8
CO—n-hexane extract (Soxhlet)	42.4 ± 0.8	6.9 ± 0.1	8.6 ± 0.2	42.1 ± 1.1
CK—n-hexane extract (Soxhlet)	40.6 ± 0.8	7.7 ± 0.1	10.5 ± 0.2	41.2 ± 0.9
